# Identification of New *Mycobacterium bovis* antigens and development of a multiplexed serological bead-immunoassay for the diagnosis of bovine tuberculosis in cattle

**DOI:** 10.1371/journal.pone.0292590

**Published:** 2023-10-09

**Authors:** Charlotte Moens, Patrice Filée, Adrien Boes, Christian Alie, François Dufrasne, Emmanuel André, Sylvie Marché, David Fretin

**Affiliations:** 1 Department of Animal Infectious Diseases, Laboratory of Veterinary Bacteriology, National Institute for Public Health (Sciensano), Brussels, Belgium; 2 Laboratory of Biochemistry and Genetics of Microorganisms, Louvain Institute of Biomolecular Science and Technology, UCLouvain, Louvain-la-Neuve, Belgium; 3 Laboratory of Immuno-Biology, CER Groupe, Aye, Belgium; 4 Department of Human Infectious Diseases, Laboratory of Viral Diseases, National Institute for Public Health (Sciensano), Brussels, Belgium; 5 Department of Microbiology, Immunology and Transplantation, Laboratory of Clinical Microbiology, KU Leuven, Leuven, Belgium; The University of Georgia, UNITED STATES

## Abstract

Serological assays for bovine tuberculosis diagnosis require the use of multiple *Mycobacterium bovis* specific antigens to ensure the detection of infected animals. In the present study, identification and selection process of antigens, based on data from published proteomic studies and involving the use of bioinformatics tools and an immuno-screening step, was firstly performed for identifying novel antigens that elicit an antibody response in *M*. *bovis* infection. Based on this approach, a panel of 10 *M*. *bovis* antigens [with known relevance (MPB70, MPB83, MPB70/83, and ESAT6/CFP10) and novel (Mb1961c, Mb1301c, Mb3871, Mb1403, Mb0592, and PE25/PPE41)] were constructed and thenused to develop a new multiplexed serological assay based on Luminex technology. The performance of the Luminex-bTB immunoassay was evaluated using sera from cattle with known tuberculosis status. Among the proteins whose ability to detect bovine tuberculosis was evaluated for the first time, PE25/PPE41 and Mb1403, but not Mb3871, showed good detection capacity. Following multiple antigen combination, the final Luminex-bTB immunoassay included seven antigens (MPB70, MPB83, MPB70/83, ESAT6/CFP10, PE25/PPE41, Mb1403, and Mb0592) and showed better global performance than the immunoassay using the four usual antigens (MPB70, MPB70/83, MPB83 and ESAT6/CFP10). The specificity and sensitivity values were, respectively, of 97.6% and 42.8% when the cut-off of two-positive antigens was used to classify samples as positive. With the use of the more-restrictive criterion of three-positive antigens, the specificity increased to 99.2% but the sensitivity decreased to 23.9%. The analysis of antigen profiles generated with the Luminex-bTB immunoassay showed that mainly serodominant proteins were recognized in samples from infected cattle. The detection of Mb1961c and Mb1301c appeared to be associated with presumed false-positive results. Moreover, sera from cattle originating from bTB-outbreaks but having inconclusive or negative skin test results were identified as positive by the Luminex-bTB immunoassay and showed an antigen pattern associated with *M*. *bovis* infection. The Luminex-bTB immunoassay including seven antigens may be useful as adjunct test for the detection of *M*. *bovis–*infected herds, and different cut-offs could be applied according to the bovine tuberculosis epidemiological context.

## 1. Introduction

Bovine tuberculosis (bTB) is an infectious chronic disease caused mainly by *Mycobacterium bovis* and dispersed widely worldwide [1]. Although its natural host is cattle, *M*. *bovis* can also infect a wide range of domestic and wild animal species, as well as humans. In Europe, the impact of this zoonotic disease on public health is limited due to the implementation of eradication and surveillance measures, such as the pasteurization or the testing and the slaughter of infected animals [2]. However, bTB remains a major economic problem, with direct and indirect costs related mainly to trade barriers for live animals and animal products, the slaughter of infected animals, and the implementation of surveillance programs [3–5].

Immune responses to *M*. *bovis* and the evolution of *M*. *bovis* infection are highly complex, rendering bTB diagnosis challenging. Cell-mediated immunity (CMI), involving mainly type 1 T helper (Th1) lymphocytes, is predominant in the early and intermediate stages of infection. Consequently, the main diagnostic tests used for bTB diagnosis, namely the single intradermal comparative cervical tuberculin (SICCT) and interferon-gamma (IFN-ɣ) tests, are based on CMI detection [4]. The humoral immune response, involving mainly Th2 lymphocytes and antibodies, develops in the late stage of the disease and can be correlated with the reduction of CMI [6,7]. Over the last two decades, several authors have reported the additional detection of an early antibody response in cattle infected experimentally with *M*. *bovis* [8–11]. Thus, the detection of the antibody response may be useful for the identification of animals with different stages of *M*. *bovis* infection, particularly animals missed by CMI-based testing. However, the kinetics of the antibody response to *M*. *bovis* antigens noticeably varies during infection and no antigen has yet been shown to be present during all stages of infection in all infected animals [12]. As a consequence, the simultaneous detection of antibody responses to a wide range of antigens with a single test could improve bTB diagnosis.

To address this problem, several multiplexed serological assays have been developed in the past two decades using different approaches, such as multi-antigen print immunoassay (MAPIA) [13,14], the Enferplex TB method [15,16], and Luminex technology [17]. In MAPIA, an air brush is used to apply multiple (up to 50) antigens to a nitrocellulose membrane in narrow bands, the strips are incubated with sera, and antigen-antibody detection is performed using a standard chromogenic method. This method continues to be used mainly to characterize antibody responses of multiple protein antigens [10,18]. The Enferplex TB chemiluminescent immunoassay enables the simultaneous detection of antibody responses to more than 20 antigens. It has been commercialized as kits for bTB detection in cattle, goats, and camelids using variable numbers of antigen [15,19,20]. In a recent field study, the Enferplex Bovine TB antibody kit employing 11 antigens and sera from cattle showed greater sensitivity than did the IDEXX enzyme-linked immunosorbent assay (ELISA) for *M*. *bovis* antibody detection using only the MPB70 and MPB83 proteins in peptide form [21]. Luminex technology enables the simultaneous, highly flexible detection of antibodies against up to 80 antigens. The antigens are coupled covalently with color-coded fluorescent bead sets mixed in the same wells. After serum incubation, antigen-antibody reactions are detected using a dual laser system based on the principle of flow cytometry [22,23]. In two recent studies, Luminex assays employing immunodominant *M*. *bovis* antigens (MPB70, MPB83, ESAT6, and CFP10) were used for bTB diagnosis in cattle [17] and pigs [24].

The aim of the present study was two-fold. Firstly, to identify and select novel seroreactive *M*. *bovis* antigens from data published reports of bovine purified protein derivative (bovine PPD) proteomic analyses [25,26]. Secondly, to develop a new multiplexed serological assay for bTB detection in cattle using Luminex technology with antigens previously selected.

## 2. Material and methods

### 2.1. Ethical approval

Ethical approval to collect the blood samples was not required for this study. Serum samples, SICCT results, and organs for bacteriology were obtained as part of Belgian national bTB control program, in compliance with official guidelines for the control of bTB in Belgium (*i*.*e*., the Federal Agency for the Safety of the Food Chain [FASFC] and veterinary services).

### 2.2. Serum samples

For the development of a negative serum sample panel, 376 serum samples obtained from cattle originating from herds in Belgium (herd prevalence < 0.1%) that had tested negative by SICCT test for the past 5 years, according to the measures established by Council Directive 64/432/EEC, were used. Whether the samples were collected within the amnestic window (*i*.*e*., 5–30 days after tuberculin injection) was not known. The samples were tested for paratuberculosis using the ID Screen^®^ paratuberculosis indirect-screening test (IDVet, Montpellier, France) as described by the manufacturer. Results for four samples were positive, according to the cut-off established by the manufacturer.

For the development of a positive serum sample panel, 366 serum samples were collected (2016–2020) from cattle in five Belgian herds considered as bTB-outbreaks (*i*.*e*. with at least one animal in herd positive by bacterial isolation for *M*. *bovis*). One hundred sixty-six, 138, and 62 samples were from animals with negative, positive (bovine PPD 4 mm > avian PPD), and inconclusive (bovine PPD 1–4 mm > avian PPD) SICCT test results, respectively. The blood samples were collected 15–30 days after the diagnostic skin test. Organs from 126 animals randomly collected during five bTB-outbreaks (regardless of SICCT results) were examined by culture and real-time polymerase chain reaction (RT-PCR) to determine the presence of *M*. *bovis*. Those from 37 (29.4%) animals showed positivity according to one or both methods, and 24 (64.9%) of those animals had gross lesions. The data were analyzed by the Sciensano veterinary bacteriology service (Brussels, Belgium), the Belgian reference laboratory for bTB diagnosis.

### 2.3. Identification of candidate proteins

To identify new potential immunogenic candidate proteins, a review of previous proteomic analyses of bovine PPDs was conducted [25,26]. The abundances of proteins identified in bovine PPDs by Roperto *et al*. [25] and Infantes-Lorenzo *et al*. [26] were given relative to normalized spectral counts and expressed as percentages. The TubercuList database (http://genolist.pasteur.fr/TubercuList/) was used to identify candidate proteins (those with *M*. *bovis* and *Mycobacterium tuberculosis* locus tags) based on published accession numbers and associated FASTA sequences. Lists of proteins identified in the PPD of *Mycobacterium avium* (avian PPD) given by Borsuk *et al*. [27] and Infantes-Lorenzo *et al*. [26] were used to identify proteins shared by avian and bovine PPDs. The Protein-BLAST (BlastP) tool available on the National Center for Biotechnology Information (NCBI, https://www.ncbi.nlm.nih.gov/) server was also used to identify homologous proteins, which shared more than 55% (arbitrary cut-off) sequence identity with members of the *M*. *avium*–*intracellulare* complex (MAC).

The subcellular localizations of proteins were predicted using SignalP 5.0 for the prediction of Sec- and Tat-dependent signal peptides for secretion [28]. SecretomeP 2.0 was used for the prediction of non-classical secreted proteins [29]. These programs are freely available from the Centre for Biological Sequence Analysis of the Technical University of Denmark (http://www.cbs.dtu.dk/services). The subcellular localizations of uncharacterized proteins were predicted using the online bioinformatics tools PSORTb 3.0 ([30]; https://psort.org/psortb/) and Gpos-mPLoc ([31]; http://www.csbio.sjtu.edu.cn/bioinf/Gpos-multi/). UniProt (http://www.uniprot.org), a freely available protein information hub, was also used to obtain information about proteins’ subcellular localizations.

### 2.4. *M*. *bovis* antigen production

The structural and biochemical parameters of candidate proteins, along with the positions of B-cell epitopes, were assessed based on their primary sequences and three-dimensional (3D) structures, when available, for the design of genetic constructions and acquisition of relevant recombinant antigens. The biochemical characteristics of the proteins were also used to guide expression, purification, and monitoring steps. A specific chaperone (the disulfide isomerase DsbC) was co-expressed with proteins containing cysteine bond(s) to promote good protein folding. Each protein was fused to a polyhistidine tag (_6_His-tag) to facilitate purification by affinity chromatography and, with the exception of the ESAT6/CFP10 heterodimer, a polylysine tag (_6_Lys-tag) to promote oriented covalent binding to carboxylated Luminex beads (**S1 Table**).

Genes encoding the candidate proteins were synthetized independently and cloned into the prokaryotic expression vector pET17b by GeneCust (Boynes, France). Clones were isolated and amplified following transformation with *Escherichia coli* BL21 (DE3) or Origami (DE3) competent cells, with or without pLys system addition (**S2 Table**). Cells were grown at 37°C until to reach the optical density (OD) at 600 nm of 0.6–0.8 and were then induced by addition of 1 mM isopropyl β-D-thiogalactopyranoside (IPTG). Growth was then continued at 25°C overnight or at 37°C for 4 h. Induced cells were pelleted by centrifugation, re-suspended in an optimal lysis buffer [50 mM phosphate buffer (pH 7.4) and 500 mM NaCl], and mechanically disrupted by sonication. Solubility was determined after bacterial lysis and gel analysis of the total, soluble, and insoluble fractions. All proteins were expressed in soluble form. Samples were clarified by centrifugation, and the supernatants were applied to a Nickel-Sepharose column for affinity purification. The elution fractions were analyzed by sodium dodecyl sulfate polyacrylamide gel electrophoresis (SDS-PAGE; BioRad, Hercules, CA, USA). Fractions containing proteins of interest were pooled and dialyzed against the storage buffer [50 mM phosphate buffer (pH 7.4) and 500 mM NaCl, with or without 10% glycerol] to eliminate imidazole. An additional purification step (size-exclusion chromatography) was applied for MPB proteins. Proteins were quantified by absorbance at 280 nm or bicinchoninic acid assay (Pierce™ BCA assay; Thermo Fisher Scientific, Waltham, MA, USA). Protein purity was estimated by densitometric analysis of the SDS-PAGE gels using ImageJ software. The final protein products were stored at –80°C until further use. Protein contents were confirmed at each step of the process by SDS-PAGE and western blotting using anti–_6_His tag antibody conjugate with alkaline phosphatase (Columbia Biosciences, Frederick, MD, USA). The following individual and fusion proteins (at the indicated concentrations and purity) were used for the immuno-screening step by ELISA or western blotting: Mb1961c (1.25 mg/ml, >90%), Mb1301c (1.91 mg/ml, >90%), MPB70 (2.4 mg/ml, >90%), MPB83 (0.28 mg/ml, >90%), MPB70/83 (0.20 mg/ml, 78%), PE25/PPE41 (0.63 mg/ml, >90%), Mb2970c (0.258 mg/ml, 57%), Mb0923 (0.14 mg/ml, 64.5%), Mb0592 (0.71 mg/ml, 69.5%), Mb3645c (0.25 mg/ml, <50%), Mb1403 (0.39 mg/ml, 64.5%), Mb1454 (0.099 mg/ml, 44.5%), Mb3871 (0.20 mg/ml, 61.5%), and Mb2659c (0.31 mg/ml, 54%). The proteins Mb3646c and Mb1300c remained non-produced. The low purities and concentrations of some proteins may be associated with weak yields or degradation. Co-expression of the ESAT-6 and CFP10 proteins was achieved through a bicistronic vector containing an internal ribosome entry-site element between the two genes encoding the proteins of interest. This approach yielded a soluble complexed form [ESAT6/CFP10 (1:1)] close to that produced naturally [32], with a good concentration (0.81 mg/ml) and >90% purity.

### 2.5. In-house indirect ELISA

Antigens were diluted to concentrations of 2.5 μg/ml (MPB83, MPB70, MPB70/83, ESAT6-CFP10) and 5 μg/ml (PE25/PPE41) in coating buffer [50 mM bicarbonate buffer (pH 9.6)]. The wells of 96-wells plates were sensitized with 100 μl antigen solution at 4°C overnight. The plates were washed four times with the wash buffer (NaCl 0.15 M, 0.01% Tween 20, and water) before blocking with phosphate-buffered saline (PBS) containing 2% bovine serum albumin (BSA) for 60 min at 37°C. After washing, they were incubated with serum samples diluted in PBS and 0.05% Tween 20 at concentrations ranging from 1:10 to 1:640 for 60 min at 37°C, then washed four times. The plates were then incubated with recombinant protein G–peroxidase conjugate (Life Technologies Europe BV, Merelbeke, Belgium) as a secondary antibody, diluted at 1:10,000 in protein G buffer [10 mM Na_2_HPO_4_ buffer, 150 mM NaCl, 0.1% Tween 80 (pH 7.2)], for 60 min at 37°C, followed by washing as described previously. Antigen-antibody reactions were revealed by the addition of 3,3′,5,5′-tetramethylbenzidine substrate solution and then stopped with 2 M H_2_SO_4_. ODs at 450 nm were measured using a BioTek spectrophotometer.

### 2.6. Western blotting

Proteins (20 μg) were loaded onto a 4–20% SDS-PAGE gel (BioRad, Hercules, CA, USA) for electrophoretic separation for 1 h at 160 V. Electrophoretic transfer of the proteins onto nitrocellulose membranes was then carried out using Transblot^®^ Turbo™ (BioRad, Hercules, CA, USA) with a transfer buffer at 25 V and 2.5 A for 11 min. The membranes were then blocked with PBS containing 0.1% Tween 20 (PBS-T) and 5% casein for 2 h at room temperature (RT) before being washed and cut into strips. Each strip was incubated with a bovine serum diluted 1:400 in PBS-T with 1% casein (dilution buffer) for 2h at RT, followed by three 5-min washes with PBS-T. The secondary antibody used was recombinant protein G–peroxidase conjugate (Life Technologies Europe BV, Merelbeke, Belgium), diluted at 1:25,000 in the dilution buffer, with 2 h incubation. Following another three washes as described above, the blots were incubated with a colorimetric development solution (Pierce™ DAB substrate; Thermo Fisher Scientific, Waltham, MA, USA) for 15 min in the dark for protein detection.

### 2.7. Carbodiimide coupling of proteins to Luminex microspheres

Coupling was performed according to the two-step carbodiimide reaction protocol provided by Luminex [33]. All washing steps were performed manually using a magnetic separator rack. All incubation steps were performed at RT, in the dark and under constant shaking. Each of the 10 selected antigens was coupled covalently to a specific MagPlex-C microbead set (Mb1961c, #12; Mb1301c, #13; MPB70, #14; MPB70/83, #15; MPB83, #18; ESAT6/CFP10, #19; PE25-PPE41, #20; Mb3871, #25; Mb1403c, #27; Mb0592, #30). To determine the optimal antigen concentration for bead coating, antigens at several concentrations (5, 10, and 20 μg/10^6^ beads) were coupled to 1,25x10^6^ carboxylated beads. Briefly, carboxylated beads were activated by incubation in 80 μl activator buffer [0.1 M NaH_2_PO_4_ (pH 6.2)] to which were added 10 μl 50-mg/ml Sulfo–N-hydroxysulfosuccinimide (NHS) solution and 10 μl 50-mg/ml 1-ethyl-3-(3-dimethylaminopropyl) carbodiimide (EDC) solution for 30 min. Then, the activated beads were washed twice and incubated for 2 h with 500 μl coupling buffer [PBS1x (pH 7.4) for PE25/PPE41 and 100 mM 2-(N-morpholino)ethanesulfonic acid (pH 6.0) for the other proteins] containing the corresponding antigens. Ethanolamine (1 M, pH 8.0) was added to the coupled bead sets for 30 min to inactivate the unreacted NHS–ester groups. The microbead concentration was determined using a cell counter and the coupled microbeads were stored in the dark at 2–8°C in storage/assay buffer [PBS1x (pH 7.4), 0.02% Tween 20, 0.1% BSA, and 0.05% sodium azide (PBS-TBN)]. Each coupling reaction was confirmed with anti–_6_His tag biotin antibody (Sigma Aldrich, St Louis, MO, USA) at concentrations ranging from 0.25 to 16 μg/ml, and the reporter streptavidin R-phycoerythrin (Life Technologies Europe BV, Merelbeke, Belgium) was used for detection.

### 2.8. Luminex-bTB multiplex immunoassay

The Luminex-bTB immunoassay was developed based on the recommendations of Luminex [33]. All incubations were performed at RT, in the dark and with constant shaking, using 96-well black flat-bottomed polystyrene plates. After each incubation, washing was performed manually with a 96-well plate magnet using 3× 300 μl washing buffer [PBS1x (pH 7.4) and 0.05% Tween 20]. Coupled beads in each set were mixed together to a final concentration of 7.5 × 10^4^ beads/ml for each bead sets using the assay buffer. Then, 50 μl of the mixture was added manually to each well, resulting in 3750 beads/well for each type of bead. The plates were incubated first for 60 min with 100 μl/well blocking solution [PBS1x (pH 7.4) and 3% BSA] and then for 60 min with 100 μl/well serum samples diluted at a 1:100 ratio in PBS-TBN. The anti-bovine immunoglobulin G biotin detection antibody (Sigma Aldrich, St Louis, MO, USA) was diluted in PBS-TBN at the final concentration of 4 μg/ml and incubated for 30min. After a wash step, the reporter streptavidin R-phycoerythrin (Life Technologies Europe BV, Merelbeke, Belgium), diluted in PBS-TBN at final concentration of 6 μg/ml, was incubated for 30min. After resuspension in PBS-TBN, the beads were analyzed using the Luminex 200 device and xPonent software (Luminex Corp., Austin, TX, USA). Each bead was identified by its signature fluorescent pattern and then analyzed to determine the median fluorescent intensity (MFI) of the reporter antibody signal for 100 beads/set/well. Doublet discriminator gates of 7500–19,000 were used to exclude bead doubles and aggregates. A blank sample (*i*.*e*., coupled bead set with all reagents except the serum sample) was included in each run to monitor the bead backgrounds (inherent fluorescence) and assay reagents. The blank did not exceed 500 MFI units. One bTB-negative and one bTB-positive reference serum sample was included in each run. Each antigen was coupled to a bead set and tested in separate wells (*i*.*e*., in monoplex assays), and the bead sets were then mixed to create the multiplex assay. MFI values were obtained for all bead sets and corrected by subtracting blank values from the sample values.

### 2.9. Antigen profile generation with the Luminex-bTB immunoassay

In the Luminex-bTB multiplex immunoassay, which has a panel of seven antigens, a serum sample that recognizes no or one antigen is deemed negative and a sample that reacts to two or more antigens is deemed positive. Thus, two to seven antigens can be recognized by antibodies from bovine sera, generating profiles of recognized antigens. In this work, these profiles are presented as binary codes (0 = negative, 1 = positive) for antigens 1 to 7 (Ag1–7; MPB70, MPB70/83, MPB83, ESAT6/CFP10, PE25/PPE41, Mb1403, and Mb0592, respectively).

### 2.10. Statistical analysis

Possible interference between sets of coupled beads was assessed by comparing MFIs detected with the monoplex and multiplex assays. The results were assessed by linear regression (yielding *R*^2^ coefficients) and Spearman correlation (yielding *r* values) analyses.

Individual specificity, sensitivity, and positivity thresholds for each antigen in the Luminex-bTB immunoassay were determined by receiver operator characteristic (ROC) curve analysis. Specificity values were assessed using the 376 serum samples from bTB free-cattle and sensitivity values were estimated relative to results from 138 samples from SICCT-positive animals that were part of confirmed bTB outbreaks. A positivity threshold was selected for each antigen to obtain the best compromise between sensitivity and specificity, with the specificity value fixed to ≥97.0%. Areas under the curve (AUCs) and associated *p* value from the ROC analysis indicated the approximate capacity of the test to discriminate positive and negative samples.

The global performance of the Luminex-bTB immunoassay was evaluated using several interpretative criteria based on the number of positive antigens detected, as described by Whelan *et al*. [34], with modulation of the specificity and sensitivity depending on the number of reactive antigens considered for the classification of samples as positive or negative. The diagnostic specificity values of the Luminex-bTB immunoassay were calculated in using 376 sera from bTB-free cattle. The diagnostic sensitivity values were estimated in relation to: (1) 138 SICCT-positive animals originating from confirmed bTB-outbreaks, and (2) 37 bacteriology-positive animals.

Agreement between MPB70 or MPB83 and MPB70/83 results in the Luminex-bTB immunoassay was assessed using the *kappa (κ)* statistic. A 2 × 2 contingency table and the chi-squared test (1 degree of freedom) were used to determine whether the sensitivity and specificity results obtained with the Luminex assay in this study differed from those reported by Fontana *et al*. [17]. The GraphPad Prism 9 software was used for all statistical analyses, and *p* < 0.05 was considered to be significant.

## 3. Results

### 3.1. Identification of *M*. *bovis* immunogenic candidate proteins

**Fig 1:**
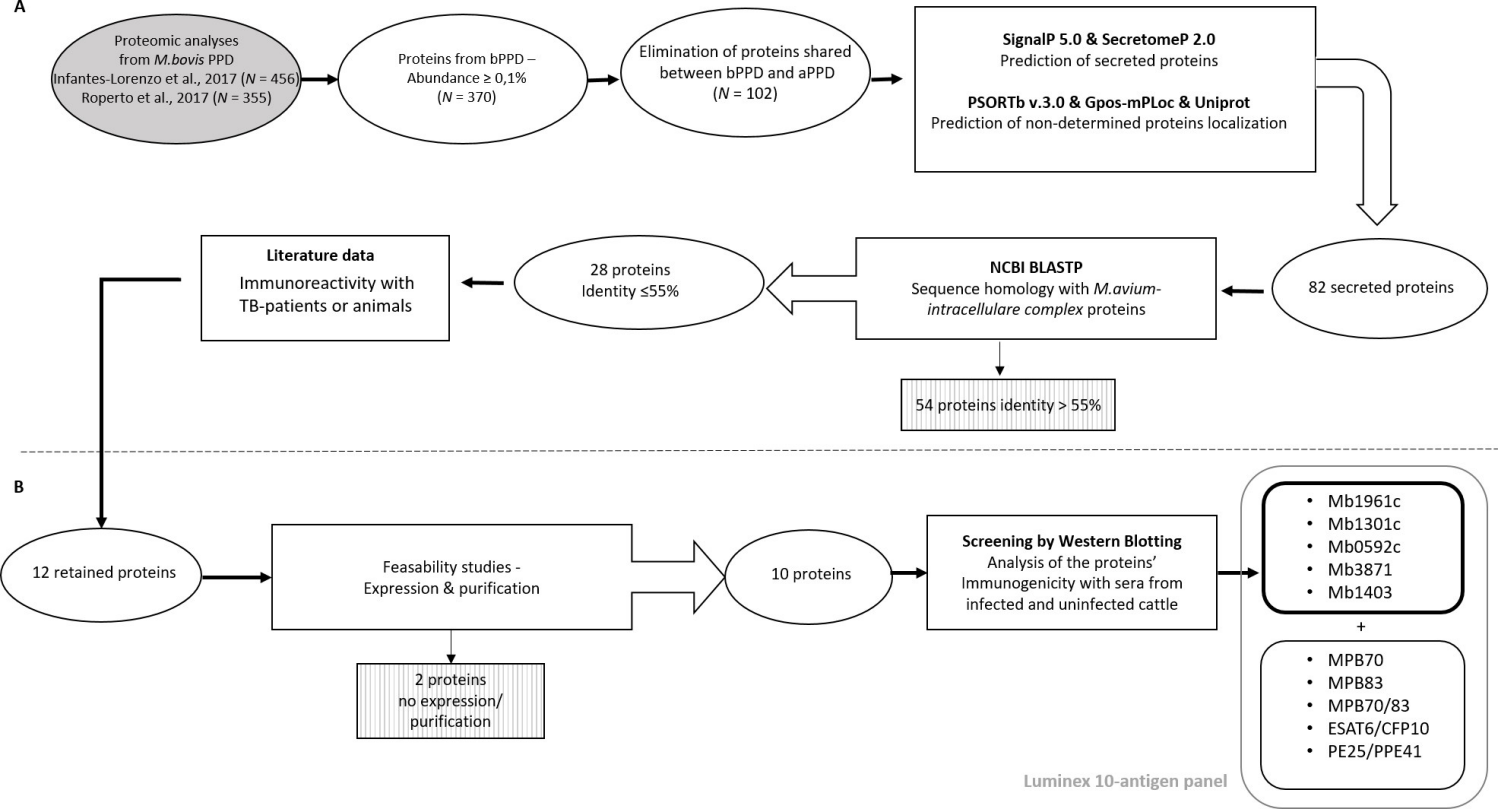
Flow of *M*. *bovis* antigen selection for use in the Luminex immunoassay development. (**A**) Selection of proteins identified from two published proteomic studies [25,26], prediction of the subcellular localization of proteins using four computer programs (SignalP, SecretomeP, PSORTb, Gpos-mPLoc) and Uniprot database, and analysis of the sequence homology of 82 potentially secreted proteins using the blastP tool. (**B**) Further characterization of 12 of 28 identified candidate proteins, with the production of 10 proteins (Mb3646c and Mb1300c remained non-produced) and screening with *M*. *bovis*–infected (n = 4) and uninfected (n = 4) sera. Mb1961c, Mb1301c, Mb0592, Mb3871, and Mb1403 were added to the base antigen panel for immunoassay development. Shaded boxes show the numbers of proteins eliminated at different steps of selection. bPPD, bovine purified protein derivative; aPPD, avian purified protein derivative.

To identify new potential immunogenic candidate proteins, we relied on the two most recent proteomic studies, conducted by Roperto *et al*. [25] and Infantes-Lorenzo *et al*. [26], in which similar methods were used to analyze bovine PPDs of different origins. Several criteria were used to select new candidate proteins (**Fig 1A**).

The abundance of proteins in bovine PPDs was one criterion used to select antigens from these two studies. Proteins with abundance ≥ 0.1%, an arbitrarily set minimum threshold, were kept (*n* = 370). To avoid the risk of cross-reactivity and thereby increase the specificity of the serological assay, proteins from bovine PPDs that were shared with avian PPDs, based on the proteomic analyses of Borsuk *et al*. [27] and Infantes-Lorenzo *et al*. [26], were removed from the candidate list. Accordingly, a total of 102 proteins with relative abundances ≥ 0.1% was found in bovine, but not avian, PPDs.

A second criterion was the subcellular localization of the candidate proteins. Particular attention was paid to secreted and outer-membrane proteins, which are more likely to stimulate an immune response based on their localizations. In total, 31 proteins were predicted to have Sec or Tat signal sequences and 35 proteins were predicted to be possibly non-classically secreted. Twelve proteins were predicted to be extracellular by the Gpos-mPLoc tool alone. Proteins Rv2626c, Rv3841, Rv3509c, and Rv0467, although not identified as secreted by the bioinformatics tools used, were recorded as potentially extracellular or secreted in the UniProt database. Thus, a total of 82 potential secreted proteins was identified. These proteins were further analyzed using Protein-BLAST to identify sequence homology with potentially cross-reacting proteins of environmental mycobacteria, including MAC members (*M*. *avium subspecies paratuberculosis*, *M*. *avium subspecies hominissuis*, *M*. *avium subspecies avium*, and *M*. *intracellulare*), broadly isolated in cattle [35,36]. Candidate proteins with <55% sequence homology with these species were selected.

Among 28 candidate proteins identified at the end of the search process, 12 new candidates were retained for further analyses, based mainly on available data (*e*.*g*., seroreactivity in patients/animals) in literature at time of this work (**Table 1**). It should be noted that among these 28 candidate proteins, four previously identified antigens, namely MPB70 (Rv2875), MPB83 (Rv2873), ESAT6 (Rv3875), and CFP10 (Rv3874), were a part of the antigen panel used in this work. In addition, based on data from the literature [37–41] and given its availability in our laboratory, we selected the PE25/PPE41 protein complex, a heterodimer secreted only by members of the *M*. *tuberculosis* complex via a specific type-VII secretion pathway [39,40]. This heterodimer was reported to induce strong humoral and cellular immune responses in patients with tuberculosis and may be involved in the reactivation of the infection [37,38,41]. To our knowledge, it has not been included previously as an antigen in an immunoassay for animal screening.

**Table 1:** Details of 12 candidate proteins selected for further evaluation in the selection process.

Rv_locus^a^	Mb_locus^b^	Protein name (characterization)	Final localization	References
Rv1926c	Mb1961c	Immunogenic protein MPB63	EC	[9,12,18]
Rv2945c	Mb2970c	Lipoprotein LppX	CM/EC	[18]
Rv0577	Mb0592	Putative glyoxylase CFP32	EC	[42,43]
Rv3841	Mb3871	Ferritin BfrB	C/CW/CM/EC	[44,45]
Rv3615c	Mb3645c	ESX-1 secretion-associated protein, EspC	C/EC	[46,47]
Rv1270c	Mb1301c	Lipoprotein LprA	CM/EC	[18]
Rv1368	Mb1403	Putative diacylated glycolipid transporter LprF	CW/CM/EC	[48]
Rv1419	Mb1454	Uncharacterized protein	M/EC	[49]
Rv0899	Mb0923	Peptidoglycan-binding protein ArfA	CW/CM/EC	[50]
Rv2626c	Mb2659c	Hypoxic response protein 1 hrp1	C/EC	[51]
Rv3616c	Mb3646c	Esx-1 secretion-associated protein, EspA	EC	[52]
Rv1269c	Mb1300c	Uncharacterized protein	CW/EC	-

^a^ Locus tag from *Mycobacterium tuberculosis* H37Rv.

^b^ Locus tag from *Mycobacterium bovis* AF2122/97.

EC, extracellular; CM, cell membrane; C, cytoplasmic; CW, cell wall; M, membrane.

### 3.2. Immunoreactivity of candidate proteins and antigen selection for Luminex-bTB immunoassay inclusion

Ten of the 12 new candidate proteins selected were efficiently expressed and purified as recombinant proteins. The immunogenicity of these 10 candidate proteins was then evaluated by western blotting using sera from *M*. *bovis*–infected (*n* = 4) and uninfected (*n* = 4) cattle. All candidate proteins except Mb1454 reacted positively to at least one of four sera from infected cattle (**Table 2**). Western blotting revealed some non-specific reactions; Mb2970c (strong band at the expected molecular weight of ~26 kDa) and Mb0592 (weak band at the expected molecular weight of ~29kDa) seemed to be recognized by antibodies from at least one serum sample from uninfected cattle. As a consequence, Mb1454 (non-reactive protein) and Mb2970c (which had a strong non-specific reaction) were removed from selection.

**Table 2:** Results of recombinant protein screening.

Rv_locus^a^	Mb_locus^b^	Molecular weight (kDa)		Western blotting results^c^									Added
			Sera from *M*. *bovis*-infected animals						Sera from negative animals				
			1		2	3	4		5	6	7	8	
Rv1926c	Mb1961c	15.7	**+**		-	-	-		-	-	-	-	Y
Rv2945c	Mb2970c	23.2	**+**		**+**	-	-		**+**	-	-	-	N
Rv0577	Mb0592	29.4	**+**		**+**	-	-		-	-	+/-	+/-	Y
Rv3841	Mb3871	7.1	**+**		-	-	-		-	-	-	-	Y
Rv3615c	Mb3645c	12.6	**+**		**+**	-	-		-	-	-	-	N
Rv1270c	Mb1301c	24.3	**+**		**+**	+/-	-		-	-	-	-	Y
Rv1368	Mb1403	24.7	**+**		+/-	+/-	-		-	-	-	-	Y
Rv1419	Mb1454	15.4	-		-	-	-		-	-	-	-	N
Rv0899	Mb0923	30.2	-		**+**	-	-		-	-	-	-	N
Rv2626c	Mb2659c	17.1	**+**		**+**	-	-		-	-	-	-	N

^a^ Locus tag from *Mycobacterium tuberculosis* H37Rv.

^b^ Locus tag from *Mycobacterium bovis* AF2122/97.

^c^ + strong band, +/- very weak band,—no band at expected molecular weight.

Sera from *M*. *bovis*-infected (1–4) and uninfected (5–8) cattle.

Y, Added; N, not added for next step.

Of the remaining 8 proteins, only those showing reactivity to at least one bTB-positive serum sample along with good expression and purification capacities were retained, for logistical and economic reasons. Accordingly, Mb1961c, Mb1301c, Mb1403, Mb3871, and Mb0592 were added to the existing panel of antigens consisting of MPB70, MPB83, MPB70/83, ESAT6/CFP10, and PE25/PPE41 to develop the Luminex bead-based immunoassay.

**Fig 2:**
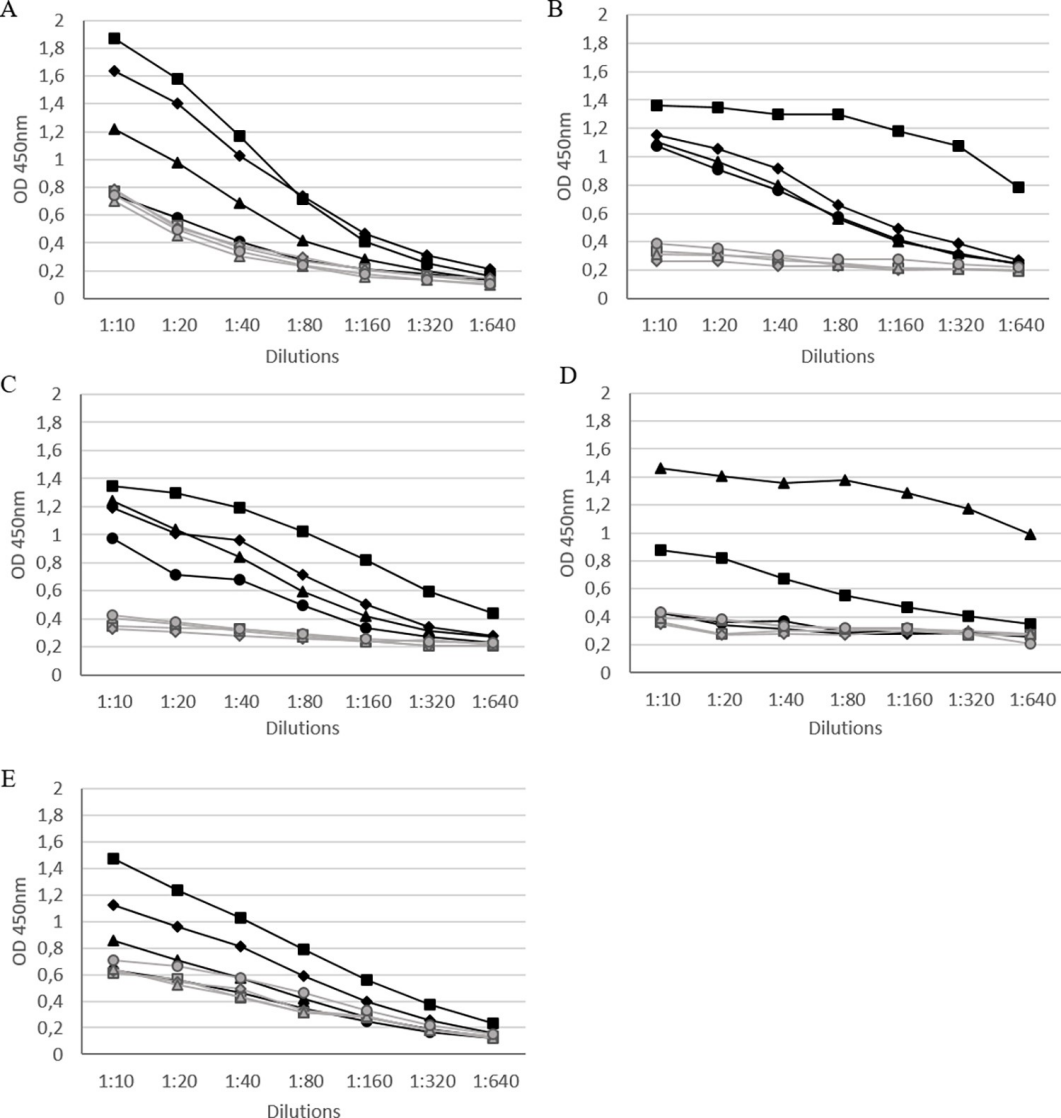
Immunoreactivity of recombinant proteins constituting the basis of the antigen panel. (A) MPB83, (B) MPB70, (C) MPB70/83 (fusion), (D) ESAT6/CFP10 (heterodimer), and (E) PE25/PPE41 (fusion) were evaluated by indirect ELISA using sera from M. bovis–infected (n = 4; black lines) and uninfected (n = 4; gray lines) cattle.

The immunoreactivity of fusion proteins (MPB70/83 and PE25/PPE41), the ESAT6/CFP10 heterodimer, and recombinant proteins (MPB70 and MPB83) produced in this study and constituting the basis of the antigen panel was also evaluated using indirect ELISA and sera from *M*. *bovis*–infected (*n* = 4) and uninfected (*n* = 4) cattle. All of them were recognized by antibodies in sera from infected cattle; PE25/PPE41 was recognized by two of four sera from infected cattle and the signal enabled distinction positive and negative sera, although more background noise was observed with uninfected bovine sera for PE25/PPE41 than for other proteins (OD 0.4–0.55 for PE25/PPE41 vs. <0.4 for other proteins at 1:40 dilution; **Fig 2**).

### 3.3. Development of the Luminex-bTB immunoassay using the selected antigens

**Fig 3:**
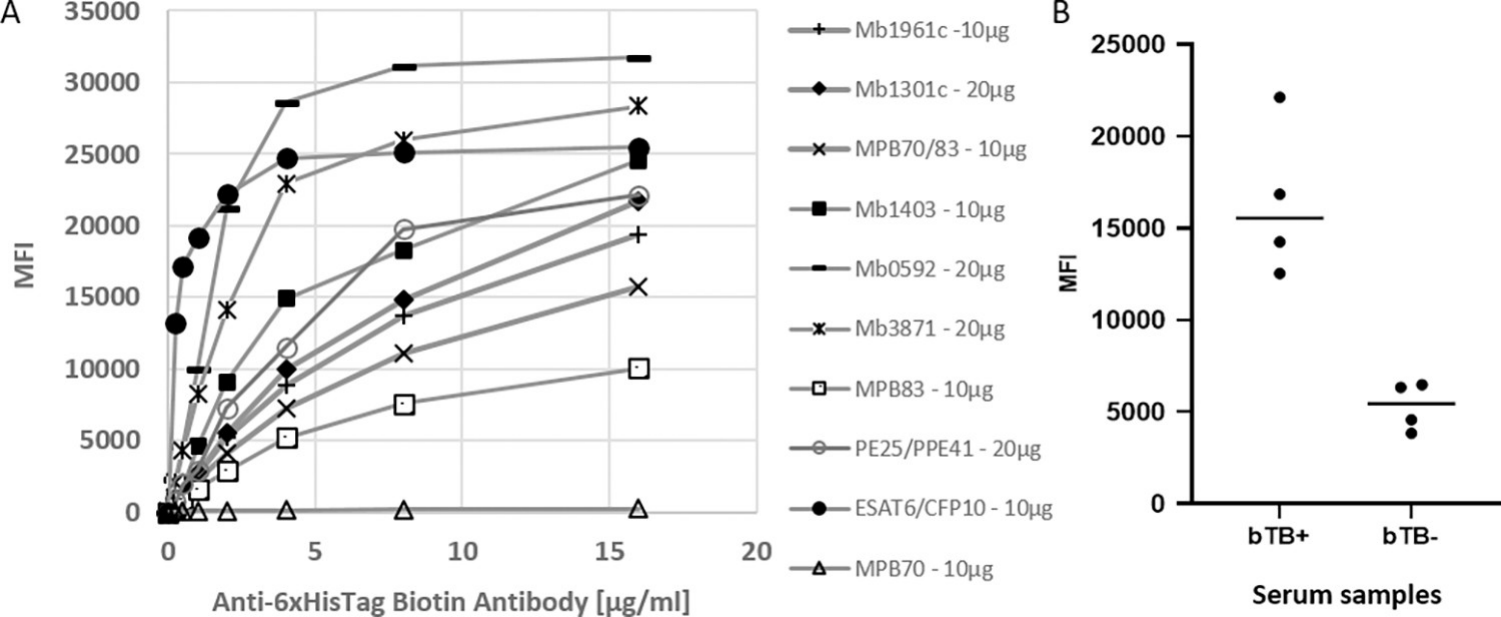
Confirmation of antigen coupling to Luminex microbeads. Confirmation was performed using (**A**) an anti-_6_His tag biotin antibody or (**B**) sera from infected (*n* = 4; bTB+) and uninfected (*n* = 4; bTB-) cattle (MPB70). MFI, median fluorescent intensity.

The optimal antigen concentrations for bead coating were determined by using several concentrations of each antigen with a fixed amount of carboxylated beads. The efficiency of coupling was then evaluated via the titration of an anti-_6_His tag biotin antibody. The optimal antigen concentrations are shown in **Fig 3A**. The anti-_6_His tag biotin antibody elicited signals from all antigens except MPB70, possibly because the protein’s coupling to the bead resulted in a conformation limiting _6_His tag antibody access. We confirmed correct coupling by testing the MPB70-coupled beads with bTB-positive and -negative serum samples at different dilutions, and the greater MFI signal was obtained with bTB-positive sera relative to bTB-negative sera at the concentration of 10 μg/10^6^ beads (**Fig 3B**). The optimal serum sample dilution for all antigen-coupled beads mixed in the same well was estimated by testing positive and negative sera at dilutions of 1:50, 1:100, and 1:200 and comparing MFIs. The optimal serum dilution was determined to be 1:100, as it yielded the largest difference between positive and negative signals and consumed least amounts of the sera.

The possibility of interference between coupled bead sets was assessed by comparing MFIs from the monoplex and multiplex assays using sera from infected and uninfected cattle. The MFIs generated in the multiplex and monoplex assays were similar for all antigens, with excellent correlation [*r* = 0.96 (Mb1301c) to *r* = 1 (MPB70, MPB83; **S1 Fig**), suggesting the absence of interference between bead sets mixed in the same well.

### 3.4. Individual antigen performance in the Luminex-bTB immunoassay

**Fig 4:**
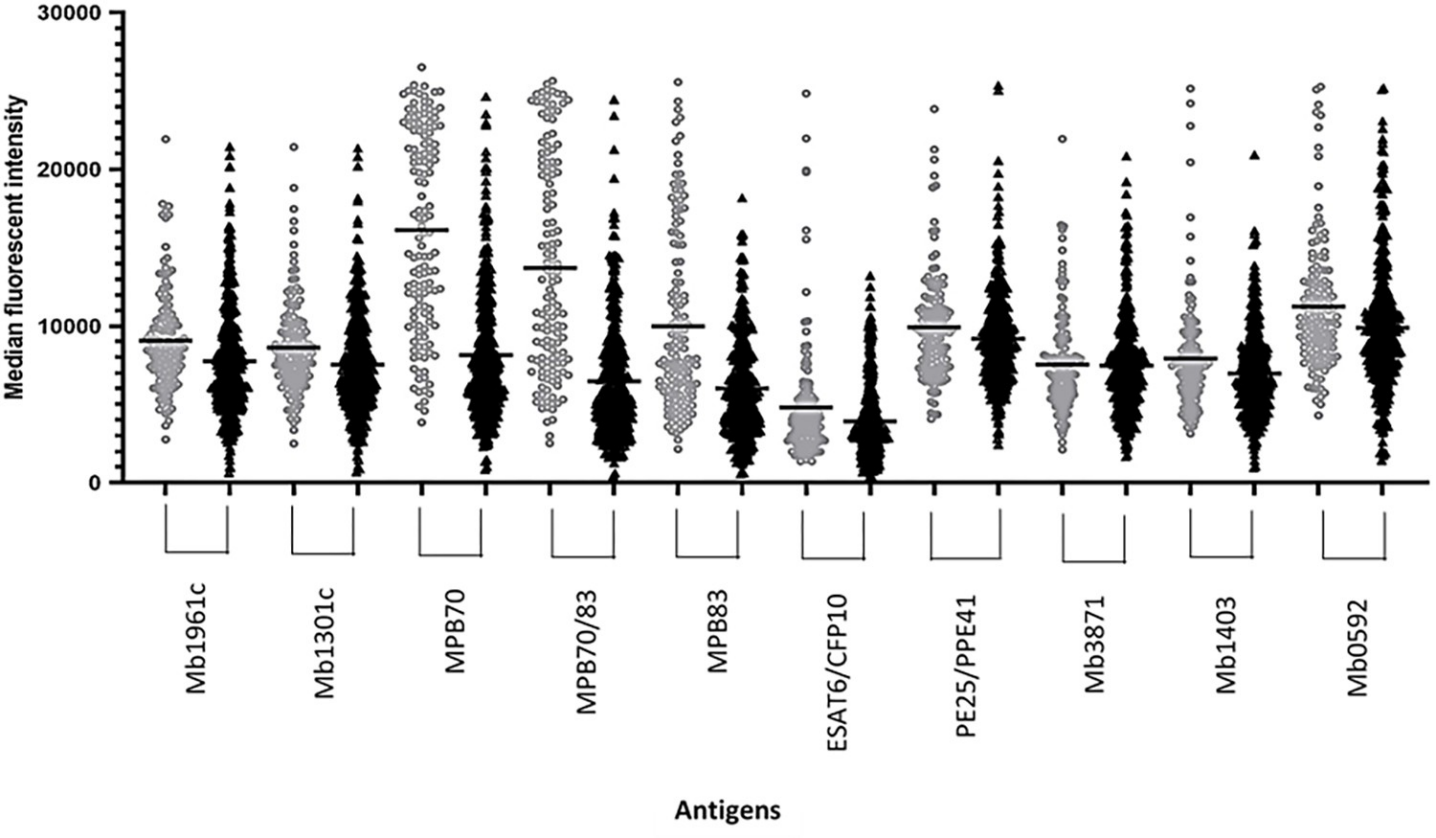
Responses of antibodies to 10 *Mycobacterium bovis* antigens used in the Luminex-bTB immunoassay. Sera from bTB-infected (*n* = 138; gray circles) and uninfected (*n* = 376; black triangles) cattle were used. Black lines indicate mean values. MFI, median fluorescent intensity.

In total, 376 bovine serum samples from Belgian bTB-free herds and 138 SICCT-positive bovine serum samples from bTB outbreaks in Belgium were screened against the panel of 10 antigens in the Luminex-bTB immunoassay (**Fig 4**).

Based on the ROC curves analysis and thresholds established, individual antigen sensitivities and specificities ranged from 5.1% to 42.7% and 97.3% to 99.5%, respectively (**Table 3**). The AUC for Mb3871 was not discriminative (AUC = 0.50; *p* = 0.93), and this antigen was removed from the multiplex reaction bead set. Thus, the Luminex-bTB immunoassay included the following nine antigens: Mb1961c, Mb1301c, MPB70, MPB70/83, MPB83, ESAT6/CFP10, PE25/PPE41, Mb1403, and Mb0592.

**Table 3:** Performance and AUC values for individual antigens in the Luminex-bTB immunoassay.

Antigen	Threshold^a^	Specificity^b^	95% CI	Sensitivity^c^	95% CI	AUC	*p*
Mb1961c	>16.500	97.9% (368/376)	95.9–98.9	5.1% (7/138)	2.5–10.1	0.64	<0.0001
Mb1301c	>14.500	97.6% (367/376)	95.5–98.7	5.1% (7/138)	2.5–10.1	0.61	<0.0001
MPB70	>18.700	97.3% (366/376)	95.2–98.5	42.7% (59/138)	34.8–51.1	0.85	<0.0001
MPB70/83	>14.500	97.3% (366/376)	95.2–98.5	42.0% (58/138)	34.1–50.4	0.83	<0.0001
MPB83	>15.500	98.9% (372/376)	97.3–99.6	21.0% (29/138)	15.0–28.5	0.72	<0.0001
ESAT6/CFP10	>12.000	99.5% (374/376)	98.1–99.9	5.1% (7/138)	2.5–10.1	0.58	<0.01
PE25/PPE41	>15.500	97.3% (366/376)	95.2–98.5	7.2% (10/138)	4.0–12.8	0.56	0.03
Mb1403	>13.500	98.1% (369/376)	96.2–99.1	5.1% (7/138)	2.5–10.1	0.58	<0.01
Mb0592	>20.500	97.9% (368/376)	95.9–98.9	5.8% (8/138)	3.0–11.0	0.60	<0.001
Mb3871	-	-	-	-	-	0.50	0.93

^a^ Expressed as median fluorescent intensity.

^b^ Specificity assessed with 376 sera from bTB-negative cattle.

^c^ Sensitivity assessed with 138 sera from SICCT-positive cattle originating from bTB-outbreaks.

AUC, area under the curve; CI, confidence interval.

The recombinant proteins MPB70 and MPB83 and the fusion protein MPB70/83 were used in this assessment. Despite moderate (MPB83 vs. MPB70/83, *κ* = 0.58) to substantial (MPB70 vs. MPB70/83, *κ* = 0.75) agreement, not all samples that responded to MPB70/83 also responded to MPB70 or MPB83, and vice versa. As a consequence, MPB70, MPB83 and the fusion protein MPB70/83 were considered as three different and independent antigens to calibrate and evaluate performance of the Luminex-bTB immunoassay

### 3.5. Luminex-bTB immunoassay performance

As shown in previous studies [16,34], multiplexed assays enable the modulation of sensitivity and specificity according to the number of reactive antigens considered to deem an animal as positive or negative. Several interpretative criteria based on the number of positive antigens (levels 1–4) detected by sera from SICCT-positive (*n* = 138), bacteriologically positive (*n* = 37), and bTB-free (*n* = 376) cattle were used to evaluate the global performance of the Luminex-bTB immunoassay.

The performance of the immunoassay including only the four major immunodominant proteins (MPB70, MPB70/83, MPB83, and ESAT6/CFP10) was evaluated, as shown in **Table 4**. To determine whether antigen addition improved the overall performance of the test, selected antigens were added individually and the assay’s performance were determined after each addition. The addition of Mb1961c or Mb1301c to the four-antigen panel did not improve the overall sensitivity of the assay, but slightly decreased its specificity according to the two-positive-antigens cut-off. In contrast, the addition of PE25/PPE41, Mb1403, or Mb0592 improved the overall sensitivity of the assay, albeit not significantly, while having no or limited impact on its specificity (**Table 4**).

**Table 4:** Global performance of the Luminex-bTB immunoassay with additions to the base four-antigen panel.

Panel	Performance	Number of positive antigens (1–4) used to classify a sample as positive			
		1	2	3	4
**4-Ag panel** ^**a**^	**Sp** ^**b**^	94.7% (356/376; CI95%: 91.9–96.5)	98.4% (370/376; CI95%: 96.6–99.3)	100.0% (376/376; CI95%: 99.0–100.0)	100.0% (376/376; CI95%: 99.0–100.0)
	**Se (vs. SICCT** ^**c**^ **)**	48.6% (67/138; CI95%: 40.4–56.8)	40.6% (56/138; CI95%: 32.7–48.9)	19.6% (27/138; CI95%: 13.8–27.0)	2.2% (3/138; CI95%: 0.7–6.2)
	**Se (vs bacteriology** ^**d**^ **)**	59.5% (22/37; CI95%: 43.5–73.6)	59.5% (22/37; CI95%: 43.5–73.6)	32.4% (12/37; CI95%: 19.6–48.5)	8.1% (3/37; CI95%:2.8–21.3)
**4Ag + PE25/PPE41**	**Sp**	92.6% (348/376; CI95%: 89.4–94.8)	97.9% (368/376; CI95%: 95.9–98.9)	100.0% (376/376; CI95%: 99.0–100.0)	100.0% (376/376; CI95%: 99.0–100.0)
	**Se (vs. SICCT)**	49.3% (68/138; CI95%: 41.1–57.5)	42.0% (58/138; CI95%: 34.1–50.4)	21.0% (29/138; CI95%: 15.0–28.5)	4.3% (6/138; CI95%: 2.0–9.2)
	**Se (vs. bacteriology)**	59.5% (22/37; CI95%: 43.5–73.6)	59.5% (22/37; CI95%: 43.5–73.6)	35.1% (13/37; CI95%: 21.8–51.2)	10.8% (4/37; CI95%: 4.3–24.7)
**4Ag + Mb1403**	**Sp**	92.8% (349/376; CI95%: 89.5–95.0)	98.4% (370/376; CI95%: 96.6–99.3)	100.0% (376/376; CI95%: 99.0–100.0)	100.0% (376/376; CI95%: 99.0–100.0)
	**Se (vs. SICCT)**	50.0% (69/138; CI95%: 41.8–58.2)	42.0% (58/138; CI95%: 34.1–50.4)	21.0% (29/138; CI95%: 15.0–28.5)	2.9% (4/138; CI95%: 1.1–7.2)
	**Se (vs. bacteriology)**	59.5% (22/37; CI95%: 43.5–73.6)	59.5% (22/37; CI95%: 43.5–73.6)	32.4% (12/37; CI95%: 19.6–48.5)	8.1% (3/37; CI95%:2.8–21.3)
**4Ag + Mb1961c**	**Sp**	93.1% (350/376; CI95%: 90.1–95.2)	97.9% (368/376; CI95%: 95.9–98.9)	100.0% (376/376; CI95%: 99.0–100.0)	100.0% (376/376; CI95%: 99.0–100.0)
	**Se (vs. SICCT)**	50.7% (70/138; CI95%: 42.5–58.9)	40.6% (56/138; CI95%: 32.7–48.9)	19.6% (27/138; CI95%: 13.8–27.0)	4.3% (6/138; CI95%: 2.0–9.2)
	**Se (vs. bacteriology)**	59.5% (22/37; CI95%: 43.5–73.6)	59.5% (22/37; CI95%: 43.5–73.6)	32.4% (12/37; CI95%: 19.6–48.5)	16.2% (6/37; CI95%:7.6–31.1)
**4Ag + Mb1301c**	**Sp**	92.8% (349/376; CI95%: 89.5–95.0)	97.9% (368/376; CI95%: 95.9–98.9)	100.0% (376/376; CI95%: 99.0–100.0)	100.0% (376/376; CI95%: 99.0–100.0)
	**Se (vs. SICCT)**	50.7% (70/138; CI95%: 42.5–58.9)	40.6% (56/138; CI95%: 32.7–48.9)	19.6% (27/138; CI95%: 13.8–27.0)	4.3% (6/138; CI95%: 2.0–9.2)
	**Se (vs. bacteriology)**	59.5% (22/37; CI95%: 43.5–73.6)	59.5% (22/37; CI95%: 43.5–73.6)	32.4% (12/37; CI95%: 19.6–48.5)	16.2% (6/37; CI95%:7.6–31.1)
**4Ag + Mb0592**	**Sp**	93.4% (351/376; CI95%: 90.4–95.5)	98.1% (369/376; CI95%: 96.2–99.1)	99.5% (374/376; CI95%: 98.1–99.8)	100.0% (376/376; CI95%: 99.0–100.0)
	**Se (vs. SICCT)**	50.7% (70/138; CI95%: 42.5–58.9)	41.3% (57/138; CI95%: 33.4–49.6)	20.3% (28/138; CI95%: 14.4–27.8)	4.3% (6/138; CI95%: 2.0–9.2)
	**Se (vs. bacteriology)**	67.6% (25/37; CI95%: 51.5–80.4)	59.5% (22/37; CI95%: 43.5–73.6)	32.4% (12/37; CI95%: 19.6–48.5)	13.5% (5/37; CI95%: 5.9–28.0)
**7-Ag panel** ^**e**^	**Sp**	89.6% (337/376; CI95%: 86.1–92.3)	97.6% (367/376; CI95%: 95.5–98.7)	99.2% (373/376; CI95%: 97.7–99.7)	100.0% (376/376; CI95%: 99.0–100.0)
	**Se (vs. SICCT)**	52.9% (73/138; CI95%: 44.6–61.0)	42.8% (59/138; CI95%: 34.8–51.1)	23.9% (33/138; CI95%: 17.6–31.7)	7.2% (10/138; CI95%: 4.0–12.8)
	**Se (vs. bacteriology)**	67.6% (25/37; CI95%: 51.5–80.4)	59.5% (22/37; CI95%: 43.5–73.6)	35.1% (13/37; CI95%: 21.8–51.2)	13.5% (5/37; CI95%: 5.9–28.0)
**9-Ag panel** ^**f**^	**Sp**	88.3% (332/376; CI95%: 84.6–91.2)	95.5% (359/376; CI95%: 92.9–97.2)	98.4% (370/376; CI95%: 96.6–99.3)	99.7% (375/376; CI95%:98.5–99.9)
	**Se (vs. SICCT)**	54.3% (75/138; CI95%: 46.0–62.5)	44.9% (62/138 ; CI95%: 36.9–53.2)	24.6% (34/138; CI95%: 18.2–32.4)	8.0% (11/138; CI95%:4.5–13.7)
	**Se (vs. bacteriology)**	67.6% (25/37; CI95%: 51.5–80.4)	59.5% (22/37; CI95%: 43.5–73.6)	35.1% (13/37; CI95%: 21.8–51.2)	16.2% (6/37; CI95%:7.6–31.1)

^a^ MPB70, MPB70/83, MPB83 and ESAT6/CFP10.

^b^ Specificity assessed with 376 sera from bTB-free cattle.

^c^ Sensitivity assessed with 138 sera from SICCT-positive cattle.

^d^ Sensitivity assessed with 37 sera from bacteriology-positive cattle.

^e^ MPB70, MPB70/83, MPB83, ESAT6/CFP10, PE25/PPE41, Mb1403, and Mb0592.

^f^ Mb1961c, Mb1301c, MPB70, MPB70/83, MPB83, ESAT6/CFP10, PE25/PPE41, Mb1403, and Mb0592.

Ag, antigen; CI, confidence interval; SICCT, single intradermal comparative cervical tuberculin test.

Among all possible antigen combinations, the Luminex-bTB immunoassay including seven antigens (MPB70, MPB70/83, MPB83, ESAT6/CFP10, PE25/PPE41, Mb1403, and Mb0592) showed the best overall performance relative to that of the nine-antigen immunoassay. The immunoassay with a seven-antigen panel was used for the rest of the present work.

With an increasing number of positive antigens, the assay’s specificity increased while its sensitivity decreased (**Table 4**). The two-positive-antigens cut-off, a standard criterion that offers a good compromise between specificity and sensitivity, was used in this work to deem a sample positive. The more stringent three-positive-antigens cut-off was considered as a criterion of high specificity of the test.

### 3.6. Profiles of antigens recognized in the Luminex-bTB immunoassay

Of the 376 serum samples from bTB-free cattle, nine reacted positively to at least two antigens of the seven-antigen immunoassay panel, yielding false-positive results. Of the 138 serum samples from SICCT-positive cattle and 37 samples from bacteriologically positive cattle, 59 and 22, respectively, reacted positively, yielding true-positive results. We analyzed the profiles of the antigens recognized by antibodies from these sera to identify any associated more with false-positive or true-positive results.

First, we determined the number of positive antigens in each population. The maximum numbers of positive antigens in sera from bTB-free and bTB-positive cattle, were three and five, respectively (**Table 5**).

**Table 5:** Profiles of antigens recognized by antibodies in sera from SICCT-positive, bacteriologically-positive and bTB-free cattle in positive responses (to two antigens or more) obtained with the Luminex-bTB immunoassay and a seven-antigen panel.

Antigen profile^a^^,^^b^	SICCT-positive cattle *(N = 59)*	Bacteriologically-positive cattle *(N = 22)*	bTB-free cattle *(N = 9)*
1100000	39.0% (23/59)	36.4% (8/22)	44.4% (4/9)
0110000	1.7% (1/59)	4.5% (1/22)	0.0% (0/9)
1000010	1.7% (1/59)	0.0% (0/22)	0.0% (0/9)
0000110	0.0% (0/59)	0.0% (0/22)	11.1% (1/9)
1000100	0.0% (0/59)	0.0% (0/22)	11.1% (1/9)
0100100	1.7% (1/59)	0.0% (0/22)	0.0% (0/9)
0001101	0.0% (0/59)	0.0% (0/22)	11.1% (1/9)
1110000	28.8% (17/59)	27.3% (6/22)	0.0% (0/9)
1100100	3.4% (2/59)	4.5% (1/22)	0.0% (0/9)
1110001	1.7% (1/59)	0.0% (0/22)	0.0% (0/9)
1110100	3.4% (2/59)	0.0% (0/22)	0.0% (0/9)
1101000	1.7% (1/59)	4.5% (1/22)	0.0% (0/9)
1100010	1.7% (1/59)	0.0% (0/22)	0.0% (0/9)
1010001	0.0% (0/59)	0.0% (0/22)	22.2% (2/9)
1110010	1.7% (1/59)	0.0% (0/22)	0.0% (0/9)
0100111	1.7% (1/59)	0.0% (0/22)	0.0% (0/9)
1101101	1.7% (1/59)	4.5% (1/22)	0.0% (0/9)
0110010	1.7% (1/59)	0.0% (0/22)	0.0% (0/9)
0101001	1.7% (1/59)	0.0% (0/22)	0.0% (0/9)
1111100	3.4% (2/59)	4.5% (1/22)	0.0% (0/9)
0111001	1.7% (1/59)	4.5% (1/22)	0.0% (0/9)
1111000	1.7% (1/59)	9.1% (2/22)	0.0% (0/9)

^a^ Binary codes (positive = 1, negative = 0) for antigens 1–7 (MPB70, MPB70/83, MPB83, ESAT6/CFP10, PE25/PPE41, Mb1403, and Mb0592).

^b^ Rare profiles unique to bTB-free cattle are underlined.

SICCT, single intradermal comparative cervical tuberculin test.

We then analyzed the profiles of antigens in false- and true-positive sera. The association of MPB70 and MPB70/83 (Ag1 and Ag2, respectively; profile 1100000), although recognized by antibodies in few sera from negative cattle, was overrepresented in bTB-positive populations. The association of three serodominant proteins [MPB70, MPB70/83, and MPB83 (Ag1–3); profile 1110000] was recognized only by antibodies in sera from bTB-positive cattle. Antigens ESAT6/CFP10 (Ag4), PE25/PPE41 (Ag5), MB1403 (Ag6), and Mb0592 (Ag7) were also recognized by antibodies in sera from SICCT-positive cattle and, except for Mb1403, those from bacteriologically positive animals, but almost always in association with at least one serodominant protein. Some rare profiles unique to false-positive sera (underlined in **Table 5**) involved Ag4–7 with or without association with serodominant proteins. Of note, the association of Mb1961c and Mb1301c, proteins not included in the final Luminex-bTB immunoassay, was represented mainly in sera from bTB-free cattle and not or only to a limited extent in sera from bTB-positive populations.

### 3.7. Evaluation of the Luminex-bTB immunoassay with samples from animals deemed negative or inconclusive by the skin test

To determine the Luminex-bTB immunoassay’s potential (including seven antigens) to detect infected cattle missed with traditional SICCT testing, sera from animals originating from bTB-outbreaks herds with inconclusive and negative SICCT-results were evaluated.

Of the 62 sera from animals with inconclusive skin test results, 25 (40.3%) yielded positive immunoassay results; 20 (32.3%) showed positivity for at least two antigens and five (8.1%) showed positivity for at least three antigens. Nine (5.4%) of 166 sera from SICCT-negative animals from *M*. *bovis*–infected herds yielded positive immunoassay results according to the two-positive-antigens cut-off, and one (0.6%) serum sample showed positivity for four antigens.

The antigen profiles observed for sera from SICCT-positive animals were also observed among sera from SICCT-inconclusive and SICCT-negative populations (**Table 6**). Two profiles, underlined in **Table 6**, were observed only for sera from SICCT-inconclusive and/or SICCT-negative animals. One of these profiles, involving positivity to MPB70 and PE25/PPE41 antigens (1000100), was recognized by sera from 2 of 20 animals with inconclusive SICCT test results and 1 of 9 presumed false-positive sera. Of note, the association of Mb1961c and Mb1301c, potentially associated with cross-reaction, was found in two sera from SICCT-negative cattle.

**Table 6:** Profiles of antigens recognized by antibodies in sera from SICCT-inconclusive and SICCT-negative cattle, originating from *M*. *bovis*-infected herds, in positive responses (to two or more antigens) obtained with the Luminex-bTB immunoassay and a seven-antigens panel.

	Sera with at least two positive antigens		
Antigen profile^a^^,^^b^	SICCT-positive cattle *(N = 59)*	SICCT-inconclusive cattle *(N = 20)*	SICCT-negative cattle *(N = 9)*
1100000	39.0% (23/59)	45.0% (9/20)	55.6% (5/9)
0110000	1.7% (1/59)	15.0% (3/20)	11.1% (1/9)
1000010	1.7% (1/59)	0.0% (0/20)	11.1% (1/9)
1000100	0.0% (0/59)	10.0% (2/20)	0.0% (0/9)
0100100	1.7% (1/59)	0.0% (0/20)	0.0% (0/9)
0100010	0.0% (0/59)	5.0% (1/20)	11.1% (1/9)
1110000	28.8% (17/59)	5.0% (1/20)	0.0% (0/9)
1100100	3.4% (2/59)	5.0% (1/20)	0.0% (0/9)
1110001	1.7% (1/59)	0.0% (0/20)	0.0% (0/9)
1110100	3.4% (2/59)	0.0% (0/20)	11.1% (1/9)
1101000	1.7% (1/59)	0.0% (0/20)	0.0% (0/9)
1100010	1.7% (1/59)	5.0% (1/20)	0.0% (0/9)
1110010	1.7% (1/59)	0.0% (0/20)	0.0% (0/9)
0100111	1.7% (1/59)	0.0% (0/20)	0.0% (0/9)
1101101	1.7% (1/59)	0.0% (0/20)	0.0% (0/9)
0110010	1.7% (1/59)	0.0% (0/20)	0.0% (0/9)
0101001	1.7% (1/59)	0.0% (0/20)	0.0% (0/9)
1111100	3.4% (2/59)	0.0% (0/20)	0.0% (0/9)
0111001	1.7% (1/59)	0.0% (0/20)	0.0% (0/9)
1111000	1.7% (1/59)	10.0% (2/20)	0.0% (0/9)

^a^ Binary codes (positive = 1, negative = 0) for antigens 1–7 (MPB70, MPB70/83, MPB83, ESAT6/CFP10, PE25/PPE41, Mb1403, and Mb0592).

^b^ Profiles only found in sera from SICCT-inconclusive and/or negative cattle are underlined.

SICCT, single intradermal comparative cervical tuberculin test.

## 4. Discussion and conclusion

Antibody response–based tests are generally easy, rapid, and inexpensive to implement, and require single blood sampling. They can be used in combination with traditional CMI-based techniques to improve bTB diagnosis. However, considering the kinetics of bTB, the use of multiple antigens may be required to detect the full spectrum of the disease, from early infection to advanced stages [12,13,53]. The multiplexed Luminex platform is used for many serological applications in veterinary medicine [54–57]. However, few studies have involved the use of this technology for the serological diagnosis of bTB [17,24]. In the present study, a panel of antigens including four major known immunogenic proteins of *M*. *bovis* (MPB70, MPB83, the MPB70/83 fusion protein, and the ESAT6/CFP10 heterodimer), the PE25/PPE41 fusion protein, and four other proteins (Mb1961c, Mb1301c, Mb1403, Mb3871, and Mb0592) was selected and used to develop and evaluate a multiplexed serological test based on Luminex technology for the detection of bTB in cattle.

The secreted protein MPB70 and cell wall lipoprotein MPB83 are highly homologous (with ~70% identity) and have unknown functions. They have been reported to induce a strong humoral response in a broad range of species infected by *M*. *bovis* [19,58–61] and are the main serodominant proteins used for bTB detection. MPB70, MPB83 and the MPB70/83 fusion protein were included in the Luminex-bTB immunoassay to improve its global detection sensitivity. As observed in the studies of Song *et al*. [62] and Lyashchenko *et al*. [63], some *M*. *bovis*–infected animals responded positively with MPB70/83 or with MPB70 and/or MPB83 only, in antibody tests, suggesting that fusion and recombinant proteins might detect overlapping, but not identical, populations of antibodies. The highly immunogenic secreted proteins ESAT6 and CFP10, which naturally form a complex involved in disease pathogenesis and virulence, have also been reported to induce antibody and CMI responses in animals of several species with *M*. *bovis* infection [10,19,32,58,64].

Additional proteins were identified during our antigen selection process and included in the development of the multiplexed assay. Mb1961c (MPB63) is an immunogenic secreted protein of unknown function that has been described to induce CMI and antibody responses in patients infected with *M*. *tuberculosis* and *M*. *bovis*–infected animals [9,12,18,26,65–67]. The glycolipoprotein Mb1301c (LprA), involved in the regulation of innate immunity as a Toll-like receptor 2 agonist [68], was reported to induce an antibody response in *M*. *bovis*–infected cattle [18]. Mb0592 (CFP32) is a secreted protein involved, as glyoxylase, in the methylglyoxal detoxification pathway [69]. It also plays a role in the initiation of adaptive immunity [70]. It has been reported to stimulate antibody responses in patients with active tuberculosis and *M*. *bovis*–infected cattle [18,42,43]. Mb3871 (Brfb), involved in iron storage, and the glycolipoprotein Mb1403 (LprF), involved in the signaling of potassium-dependent osmotic stress, have been found to elicit antibody reactions in sera from tuberculous patients [44,45,48,71,72]. PE25 (Mb2457c) and PPE41 (Mb2456c) are secreted proteins that naturally form a complex. This dimer, involved in mycobacteria dissemination and the regulation of the immune response [38,41], induces humoral and cellular immune responses in patients infected with *M*. *tuberculosis* [37].

PE25/PPE41, Mb1403, and Mb3871, detected in sera from patients with tuberculosis [37,44,45,48], have not, to our knowledge, previously been evaluated as antigens for bTB detection with sera from *M*. *bovis*–infected cattle. With ELISA and western blotting, we demonstrated that they are also recognized by antibodies from sera from cattle naturally infected with *M*. *bovis* and may be of interest for the serological diagnosis of bTB. However, in the multiplex test, Mb3871 did not show the ability to discriminate positive and negative samples and was thus excluded from the antigen panel used for the Luminex-bTB immunoassay. In contrast, PE25/PPE41 and Mb1403 showed good capacities to discriminate samples from SICCT-positive and bTB-free cattle. The individual addition of these proteins to the panel of four major antigens improved the global sensitivity of the assay without altering its specificity, confirming that these newly identified antigens are of interest for bTB diagnosis. While both proteins were recognized by antibodies from sera from SICCT-positive cattle, PE25/PPE41, but not Mb1403, was recognized by antibodies in sera from bacteriologically positive cattle, suggesting that it represents a broader range of infection stages than does Mb1403. Additional studies are needed to confirm these results.

A bTB detection method based on Luminex technology and including the MPB70, MPB83, ESAT6, and CFP10 antigens was previously developed and evaluated [17]. The base Luminex immunoassay with a similar panel of antigens (*i*.*e*., MPB70, MPB70/83, MPB83 and ESAT6/CFP10), evaluated in the present work, showed significantly less specificity than that reported by Fontana *et al*. [17] (98.4% versus 100.0%, *p* < 0.001). Differences in antigen conformation (fusion/recombinant), populations of bTB-negative cattle, and the number of samples used (861 vs. 376 sera in the current study) may explain this discrepancy. Of note, the SICCT status (boosted or non-boosted) of bTB-free cattle was unknown in both studies [17], preventing the determination of whether the timing of blood sampling (within or outside of the anamnestic window) influences assay specificity and could explain the difference in findings. However, recent data suggest that this timing does not influence the specificity of serological tests [15].

The stages of infection detected, but also the number of cross-reactions occurring, can be expected to increase with the number of antigens included in a multiplex assay, leading to reduced specificity. The use of seven antigens (*i*.*e*. four major antigens + PE25/PPE41, Mb1403 and Mb0592) improved the sensitivity of the Luminex-bTB immunoassay developed in this study without significantly decreasing its global specificity relative to that of the four-antigen panel [97.6% vs. 98.4% (*p* = 0.43) with the two-antigen cut-off; 99.2% vs. 100.0% (*p* = 0.08) with the three-antigen cut-off]. In contrast, the use of a nine-antigen panel (*i*.*e*., addition of Mb1961c and Mb1301c) significantly decreased the specificity of the assay relative to the four-antigen panel [95.5% vs. 98.4% (*p* = 0.02) with the two-antigen cut-off; 98.4% vs. 100.0% (*p* = 0.002) with the three-antigen cut-off] without improving the assay’s sensitivity.

The presumed false-positive reactions that reduced assay specificity in the current study may be associated with the exposure of cattle to non-tuberculous mycobacteria (NTM). Species of the genus *Mycobacterium* are closely related and can share homologous proteins, resulting in antibody cross-reactivity and false-positive detection. In this study, we paid particular attention to this possibility, selecting antigens with low degrees of homology with mycobacteria from the MAC. However, likely serologically cross-reactive NTM that are not members of the MAC, such as *Mycobacterium nonchromogenicum* and *Mycobacterium kansasii*, have been isolated from cattle. The prevalence of environmental mycobacteria is highly variable according to the geographic area and identification method used [35,36,73–75]. All of the nine proteins evaluated in this work with the Luminex-bTB immunoassay, showed low degrees (<50%) of sequence homology with *M*. *nonchromogenicum* proteins. Gcebe *et al*. [76], however, identified homologous proteins of Mb1961c and Mb1301c in PPDs of *Mycobacterium fortuitum* and *M*. *nonchromogenicum*. Except for PE25 and PPE41, all proteins used in this work showed high degrees (>60%) of sequence identity with *M*. *kansasii*. Mb1301c and Mb1403, but not Mb1961c, were found in the PPD of *M*. *kansasii* [76]. Waters *et al*. [60] showed that high doses of *M*. *kansasii* in cattle may generate false-positive reactions with MPB83 and MPB70, which have high degrees (>75%) of sequence identity with this species. No data on the seroreactivity of these proteins in animals infected with these NTM or the presence of these NTM in cattle whose sera were examined in this work are available. Additional analyses should be performed to evaluate potential cross-reactions of antigens included in the Luminex-bTB immunoassay with environmental mycobacteria. Of note, although most proteins showed >90% purity in this work, the purity of proteins produced in *E*. *coli* may also impact the specificity of the assay.

The sensitivity of the Luminex-bTB immunoassay was estimated in this work relative to the sera from SICCT-positive animals originating from herds with culture-confirmed *M*. *bovis* infection. The sensitivity of the four-antigen panel obtained using the two-positive-antigens cut-off was lesser, although not significantly so (40.6% vs. 50.8%, *p* = 0.18), than that reported by Fontana *et al*. [17], who used intradermal tuberculin and/or IFN-ɣ positivity as the reference. When sera from bacteriologically positive animals were tested, the sensitivity of both assays increased (to 59.5% and 51.2%, respectively; *p* = 0.37). These results are consistent with the strong antibody response in serological tests of sera from animals with advanced bTB, particularly those with gross lesions [7,77].

Relative to that of the immunoassay with a four-antigen panel, the sensitivity (vs. SICCT positivity) of the Luminex-bTB immunoassay including seven antigens was 2.2% greater with the two-positive-antigens cut-off and 4.3% greater with the three-positive-antigens cut-off. This difference was attenuated when bacteriological positivity was used as the reference. These results suggest that the inclusion of more antigens increases the likelihood that the assay will detect SICCT-positive animals at different stages of infection. A SICCT-positive result, based on a CMI response, may reflect an animal to an early stage of infection but can also indicate a later stage of the disease, with or without visible lesions and bTB symptoms [6]. Moreover, the probability of serodominant protein detection increases with disease progression [78,79], which can reduce the difference observed between sensitivity values. It should, however, be noted that the skin test–related anamnestic effect is reflected in the sera used in this study to determine the sensitivity of the Luminex-bTB immunoassay [80,81]. Thus, the assay’s sensitivity is expected to be lesser against reference samples from cattle who have not been boosted by skin testing.

Several parameters intrinsic to Luminex technology may also have influenced the performance of the Luminex-bTB immunoassay. Variability in protein–bead coupling may alter performance, in particular the sensitivity, of Luminex tests [82]. In this work, _6_Lys and _6_His tags were included in the recombinant protein designs to enhance antigen–bead binding, as observed for the PE25/PPE41 dimer with and without the _6_Lys tag, along with the confirmation of coupling. The antigen design and production methods may lead to differences in protein conformation following binding to the 3D beads, and thus potential differences in antigenic epitopes presentation, explaining variability in assay performance among studies. Moreover, the absence of a standard method for the analysis of data produced with multi-antigen Luminex methods [17,83–86] may result in variation in specificity and sensitivity results obtained in different studies.

The analysis of the profiles of antigens recognized by antibodies from sera tested in this study showed that the recognition of true-positive sera involved the recognition of at least one serodominant protein (*i*.*e*., MPB70, MPB70/83, and/or MPB83). The other proteins included in the multiplexed assay were rarely identified without the recognition of at least one serodominant protein in samples from positive cattle populations. The antibody responses elicited by MPB70 and MPB83 have been found to be boosted by tuberculin injection due to the anamnestic effect, but whether this phenomenon can be applied to other antigens is not clear [81,87]. The association of Mb1961c and Mb1301c was identified more in samples from the negative population of cattle than in those from the positive population. We cannot exclude the possibility that these antigens are detected at stages of infection other than those represented in the samples from infected animals tested in this work.

Whelan *et al*. [34] and Fontana *et al*. [17] reported that multiplexed serological assays can be useful for the detection of bTB in skin-tested non-reactive cattle. In this study, the Luminex-bTB immunoassay identified 20 and 9 positive reactions out 62 and 166 sera from SICCT-inconclusive and SICCT-negative animals, respectively, originating from bTB-outbreaks. Although the infection status of these animals was not confirmed, most samples had an antigen profile previously associated with infection, suggesting that these animals were in the anergic stage of *M*. *bovis* infection.

Researchers have shown interest in the use of multiple antigens for bTB diagnosis in animals [17,19,21,80]. Immunodominant proteins (MPB70, MPB83, ESAT6, and CFP10) are among the most abundant proteins in bovine PPDs [25,26] and induce strong humoral responses in *M*. *bovis*–infected animals. For this reason, major serological assays are based on these antigens [15,60]. To date, no new antigen capable of inducing such a strong serological reaction, in a significant number of animals has been reported. In the present study, the addition of three new antigens (PE25/PPE41, Mb1403, and Mb0592) to a multiplexed test based on the known major antigens improved the test’s detection capacity. However, an optimizing of the test in terms of performance, mainly sensitivity, might be required to use the Luminex-bTB immunoassay in the bTB control. One reason for the major interest in Luminex technology is its flexibility, which enables target addition or removal when required. As a consequence, additional proteins could be identified, and their diagnostic contributions when included in the present Luminex-bTB immunoassay could be evaluated, in the future. In addition, the assay’s performance can be modulated easily according to situational requirements (*i*.*e*., for high specificity or high sensitivity) by altering the number of reactive antigens considered for the classification of results as positive or negative. The most stringent interpretative criterion could be used in countries where the prevalence of bTB is low and where a high degree of specificity is essential. This immunoassay could thus, subject to optimization, be applied in combination with CMI-based assays to detect infected herds, mainly those in the anergic stage of bTB, which are sources of disease maintenance and propagation.

## Supporting information

S1 Fig Analysis of interference between coupled bead sets. Correlations of median fluorescence intensities from multiplex and monoplex assays performed with 11 serum samples (six from infected and five from uninfected cattle). *R*^*2*^, linear regression coefficient; *r*, non-parametric Spearman correlation coefficient.
(TIF)

S1 Table Amino acid sequences of proteins produced. The polyhistine tags, polylysine tags, linkers, and mutations are colored red, green, blue and yellow, respectively. Accession numbers are from the Uniprot database.
(DOCX)

S2 Table Conditions of production for candidate proteins. (DOCX)
