# Supplementary figures and images for: Identification of New Mycobacterium bovis antigens and development of a multiplexed serological bead-immunoassay for the diagnosis of bovine tuberculosis in cattle
Source: PLoS One. 2023 Oct 9;18(10):e0292590. doi: 10.1371/journal.pone.0292590 (PMC10561873; doi:10.1371/journal.pone.0292590)

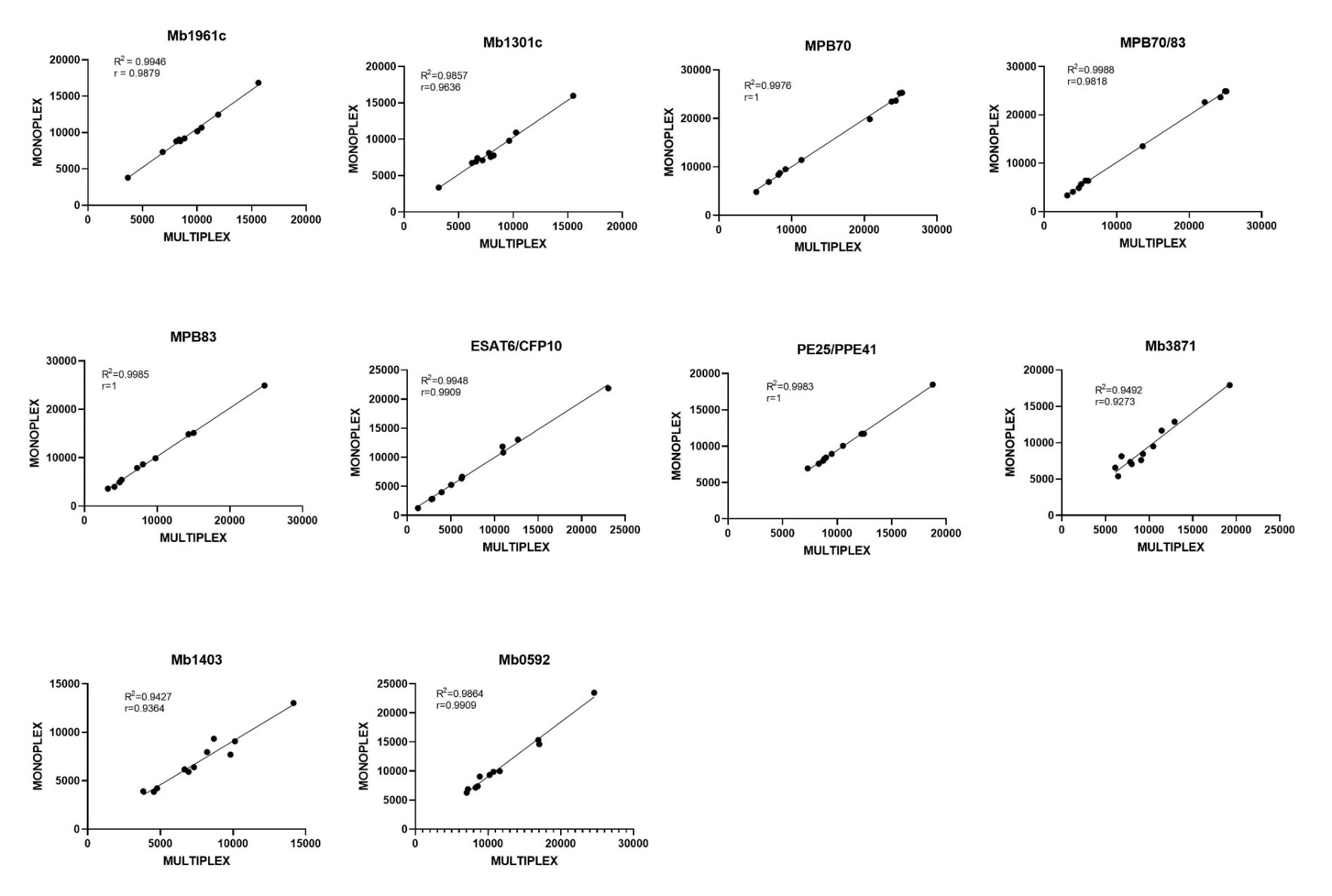

Supplement: S1 Fig — Correlations of median fluorescence intensities from multiplex and monoplex assays performed with 11 serum samples (six from infected and five from uninfected cattle). R2, linear regression coefficient; r, non-parametric Spearman correlation coefficient. (TIF) [file pone.0292590.s001.tif]
