# Supplementary material for: Identification of New Mycobacterium bovis antigens and development of a multiplexed serological bead-immunoassay for the diagnosis of bovine tuberculosis in cattle
Source: PLoS One. 2023 Oct 9;18(10):e0292590. doi: 10.1371/journal.pone.0292590 (PMC10561873; doi:10.1371/journal.pone.0292590)
Supplement: S1 Table — The polyhistine tags, polylysine tags, linkers, and mutations are colored red, green, blue and yellow, respectively. Accession numbers are from the Uniprot database. (DOCX) [file pone.0292590.s002.docx]

**S1 Table. Amino acid sequences of proteins produced.** The polyhistine tags, polylysine tags, linkers, and mutations are colored red, green, blue and yellow, respectively. Accession numbers are from the Uniprot database.

| **Protein**  **(Accession no.)** | **Amino acid Sequence** |
| --- | --- |
| MPB83 protein  (P0CAX7) | MA**HHHHHH**DLIGRGCAQYAAQNPTGPGSVAGMAQDPVATAASNNPMLSTLTSALSGKLNPDVNLVDTLNGGEYTVFAPTNAAFDKLPAATIDQLKTDAKLLSSILTYHVIAGQASPSRIDGTHQTLQGADLTVIGARDDLMVNNAGLVCGGVHTANATVYMIDTVLMPPAQKL**KKKKKK** |
| MPB70 protein  (P0A669) | MA**HHHHHH**KLGDLVGPGCAEYAAANPTGPASVQGMSQDPVAVAASNNPELTTLTAALSGQLNPQVNLVDTLNSGQYTVFAPTNAAFSKLPASTIDELKTNSSLLTSILTYHVVAGQTSPANVVGTRQTLQGASVTVTGQGNSLKVGNADVVCGGVSTANATVYMIDSVLMPPAKL**KKKKKK** |
| MPB70/83  fusion protein | MA**HHHHHH**DLIGRGCAQYAAQNPTGPGSVAGMAQDPVATAASNNPMLSTLTSALSGKLNPDVNLVDTLNGGEYTVFAPTNAAFDKLPAATIDQLKTDAKLLSSILTYHVIAGQASPSRIDGTHQTLQGADLTVIGARDDLMVNNAGLVCGGVHTANATVYMIDTVLMPPA**QKL**GDLVGPGCAEYAAANPTGPASVQGMSQDPVAVAASNNPELTTLTAALSGQLNPQVNLVDTLNSGQYTVFAPTNAAFSKLPASTIDELKTNSSLLTSILTYHVVAGQTSPANVVGTRQTLQGASVTVTGQGNSLKVGNADVVCGGVSTANATVYMIDSVLMPPAKL**KKKKKK** |
| PPE41(Q79FE1)  /PE25 (I6X486)  fusion protein | MHFEAYPPEVNSANIYAGPGPDSMLAAARAWRSLDVEMTAVQRSFNRTLLSLMDAWAGPVVMQLMEAAKPFVRWLTDLCVQLSEVERQIHEIVRAYEWAHHDMVPLAQIYNNRAERQILIDNNALGQFTAQIADLDQEYDDFWDEDGEVMRDYRLRVSDALSKLTPWKAPPPIAHSTVLVAPVSPSTASSRTDT**GGGGSHHHHHHGGGGS**MSFVITNPEALTVAATEVRRIRDRAIQSDAQVAPMTTAVRPPAADLVSEKAATFLVEYARKYRQTIAAAAVVLEEFAHALTTGADKYATAEADNIKTFS**KKKKKK** |
| ESAT6 (P0A565)/CFP10 (P0A567) heterodimer | >CFP10  MAEMKTDAATLAQEAGNFERISGDLKTQIDQVESTAGSLQGQWRGAAGTAAQAAVVRFQEAANKQKQELDEISTNIRQAGVQYSRADEEQQQALSSQMGF  >ESAT6  MTEQQWNFAGIEAAASAIQGNVTSIHSLLDEGKQSLTKLAAAWGGSGSEAYQGVQQKWDATATELNNALQNLARTISEAGQAMASTEGNVTGMFA**HHHHHH** |
| Mb0592 protein  (P9WIR3) | MA**HHHHHH**MPKRSEYRQGTPNWVDLQTTDQSAAKKFYTSLFGWGYDDNPVPGGGGVYSMATLNGEAVAAIAPMPPGAPEGMPPIWNTYIAVDDVDAVVDKVVPGGGQVMMPAFDIGDAGRMSFITDPTGAAVGLWQANRHIGATLVNETGTLIWNELLTDKPDLALAFYEAVVGLTHSSMEIAAGQNYRVLKAGDAEVGGCMEPPMPGVPNHWHVYFAVDDADATAAKAAAAGGQVIAEPADIPSVGRFAVLSDPQGAIFSVLKPAPQQ**SSGKKKKKK** |
| Ecto-domain of the Mb0923 protein (P65594) | MA**KKKKKK**G**HHHHHH**ERPQSVTGPTGVLPTLTPTSTRGASALSLSLLSISRSGNTVTLIGDFPDEAAKAALMTALNGLLAPGVNVIDQIHVDPVVRSLDFSSAEPVFTASVPIPDFGLKVERDTVTLTGTAPSSEHKDAVKRAATSTWPDMKIVNNIEVTGQAPPGPPASGPCADLQSAINAVTGGPIAFGNDGASLIPADYEILNRVADKLKACPDARVTINGYTDNTGSEGINIPLSAQRAKIVADYLVARGVAGDHIATVGLGSVNPIASNATPEGRAKNRRVEIVVN |
| Mature Mb1300c protein (P0A5E2) | MA**HHHHHH**ADVYGAIAYSGNGSWGRSWDYPTRAAAEATAVKSCGYSDCKVLTSFTACGAVAANDRAYQGGVGPTLAAAMKDALTKLGGGYIDTWACN**KKKKKK** |
| Mature Mb1301 protein (Q7U094) | MA**HHHHHH**STEGDAGKASDTAATASNGDAAMLLKQATDAMRKVTGMHVRLAVTGDVPNLRVTKLEGDISNTPQTVATGSATLLVGNKSEDAKFVYVDGHLYSDLGQPGTYTDFGNGTSIYNVSVLLDPNKGLANLLANLKDASVAGSQQADGVATTKITGNSSADDIATLAGSRLTSEDVKTVPTTVWIASDGSSHLV  QIQIAPTKDTSVTLTMSDWGKQVTATKPV**KKKKKK** |
| Mature Mb1403 protein (P65315) | MA**HHHHHH**GKKPTTASSPSPGSPSPEAQQILQDSSKATKGLHSVHVVVTVNNLSTLPFESVDADVTN  QPQGNGQAVGNAKVRMKPNTPVVATEFLVTNKTMYTKRGGDYVSVGPAEKIYDPGIILDKDRGLGA  VVGQVQNPTIQGRDAIDGLATVKVSGTIDAAVIDPIVPQLGKGGGRLPITLWIVDTNASTPAPAANLVR  MVIDKDQGNVDITLSNWGAPVTIPNPAG**KKKKKK** |
| Mature Mb1454 protein (P9WLX9) | MA**HHHHHH**DGPVQLKSRLGDVCLDAPSGSWFSPLVINPCNGTDFQRWNLTDDRQVESVAFPGECVNIGNALWARLQPCVNWISQHWTVQPDGLVKSDLDACLTVLGGPDPGTWVSTRWCDPNAPDQQWDSVP**KKKKKK** |
| Mature Mb1961c protein (P0A5Q3) | MA**HHHHHH**AYPITGKLGSELTMTDTVGQVVLGWKVSDLKSSTAVIPGYPVAGQVWEATATVNAIRGVTPAVSQFNARTADGINYRVLWQAAGPDTISGATIPQGEQSTGKIYFDVTGPSPTIVAMNNGMEDLLIWEP**SSGKKKKKK** |
| Mb2659c protein (P9WJA3) | MTTARDIMNAGVTCVGEHETLTAAAQYMREHDIGALPICGDDDRLHGMLTDRDIVIKGLAAGLDPNTATAGELARDSIYYVDANASIQEMLNVMEEHQVRRVPVISEHRLVGIVTEADIARHLPEHAIVQFVKAI**S**SPMALAS**HHHHHH**G**KKKKKK** |
| Mature Mb2970c  (P65307) | MA**HHHHHH**SSPKPDAEEQGVPVSPTASDPALLAEIRQSLDATKGLTSVHVAVRTTGKVDSLLGITSADVDVRANPLAAKGVCTYNDEQGVPFRVQGDNISVKLFDDWSNLGSISELSTSRVLDPAAGVTQLLSGVTNLQAQGTEVIDGISTTKITGTIPASSVKMLDPGAKSARPATVWIAQDGSHHLVRASIDLGSGSIQLTQSKWNEPVNVD**KKKKKK** |
| Mb3645c protein (P9WJD7) | MA**HHHHHH**MTENLTVQPERLGVLASHHDNAAVDASSGVEAAAGLGESVAITHGPYCSQFNDTLNVYLTAHNALGSSLHTAGVDLAKSLRIAAKIYSEADEAWRKAIDGLFT**KKKKKK** |
| Mb3646c protein  (A0A1R3Y4Q8) | MA**HHHHHH**MSRAFIIDPTISAIDGLYDLLGIGIPNQGGILYSSLEYFEKALEELAAAFPGDGWLGSAADKYAGKNRNHVNFFQELADLDRQLISLIHDQANAVQTTRDILEGAKKGLEFVRPVAVDLTYIPVVGHALSAAFQAPFCAGAMAVVGGALAYLAVKTLINATQLLKLLAKLAELVAAAIADIISDVADIIKGILGEVWEFITNALNGLKELWDKLTGWVTGLFSRGWSNLESFFAGVPGLTGATSGLSQVTGLFGAAGLSASSGLAHADSLASSASLPALAGIGGGSGFGGLPSLAQVHAASTRQALRPRADGPVGAAAEQVGGQSQLVSAQGSQGMGGPVGMGGMHPSSGASKGTTTKKYSEGAAAGTEDAERAPVEADAGGGQKVLVRNVV**KKKKKK** |
| Synthetic Mb3871 protein (P9WNE5) | MA**HHHHHH**DRDLRVEIPGVDTVRNQFDR**GGGGSGGGS**DRDLRVEIPGVDTVRNQFDR**KKKKKK** |
