# Supplementary material for: Identification of New Mycobacterium bovis antigens and development of a multiplexed serological bead-immunoassay for the diagnosis of bovine tuberculosis in cattle
Source: PLoS One. 2023 Oct 9;18(10):e0292590. doi: 10.1371/journal.pone.0292590 (PMC10561873; doi:10.1371/journal.pone.0292590)
Supplement: S2 Table — (DOCX) [file pone.0292590.s003.docx]

**S2 Table**. **Conditions of production for candidate proteins.**

| **Protein** | ***E. coli* strain** | **Induction condition** |
| --- | --- | --- |
| MPB83 | Origami B (DE3) pLys | 25°C, overnight |
| MPB70 | Origami B (DE3) pLys | 25°C, overnight |
| MPB70/83 | Origami B (DE3) pLys | 25°C, overnight |
| PPE41/PE25 | BL21 (DE3) | 37°C, 4h |
| ESAT6/CFP10 | BL21 (DE3) pLys | 37°C, 4h |
| Mb0592 | BL21 (DE3) | 37°C, 4h |
| Mb0923 | Origami B (DE3) | 37°C, 4h |
| Mb1300c | No transformant | - |
| Mb1301c | BL21 (DE3) | 37°C, 4h |
| Mb1403 | BL21 (DE3) | 37°C, 4h |
| Mb1454 | Origami B (DE3) | 37°C, 4h |
| Mb1961c | BL21 (DE3) | 37°C, 4h |
| Mb2659c | Origami B (DE3) | 25°C, overnight |
| Mb2970c | BL21 (DE3) | 37°C, 4h |
| Mb3645c | BL21 (DE3) pLys | 25°C, overnight |
| Mb3646c | No transformant | - |
| Mb3871 | BL21 (DE3) pLys | 37°C, 4h |
